# Serosurvey of *Trypanosoma cruzi* in persons experiencing homelessness and shelter workers of Brazil

**DOI:** 10.3389/fpubh.2023.1125028

**Published:** 2023-03-03

**Authors:** Louise Bach Kmetiuk, Gustavo Gonçalves, Anahi Chechia Do Couto, Alexander Welker Biondo, Fabiano Borges Figueiredo

**Affiliations:** ^1^Laboratory of Cell Biology, Carlos Chagas Institute, Oswaldo Cruz Foundation, Curitiba, Paraná, Brazil; ^2^Department of Veterinary Medicine, Federal University of Paraná State, Curitiba, Paraná, Brazil

**Keywords:** Chagas disease, epidemiology, health inequalities, homeless people, vulnerability

## Abstract

Although Chagas disease, caused by *Trypanosoma cruzi*, has been associated with social vulnerability worldwide, producing disability and mortality, no study to date has assessed this protozoal infection in persons experiencing homelessness. Accordingly, the present study aimed to assess anti-*T. cruzi* antibodies by Wiener Chagatest ELISA recombinant v.3.0 in serum samples of persons experiencing homelessness and related shelter workers in São Paulo, a city with reported vectors but no recent autochthonous case report. Overall, seropositivity to *T. cruzi* resulted in three of 203 (1.5%) persons experiencing homelessness and two of 87 (2.3%) shelter workers, with similar seroprevalence likely associated with their past social vulnerability. Although the seropositivity in persons experiencing homelessness and shelter workers was within 0 to 25.1% seroprevalence for chronic Chagas disease in the general Brazilian population, the disease has almost decreased 2-fold from the 1980s to 2000s, and such a wide range may not reflect the local disease status. In addition, the authors hypothesized that the similar seroprevalence and exposure between homeless persons and shelter workers herein may be more associated with shared past and present low-income social vulnerability than migratory movements, which may also include infection by sharing injecting drugs, vertical transmission, or blood transfusion. Thus, future studies are needed to confirm the active transmission of Chagas disease in São Paulo city. Moreover, Chagas disease should be considered as differential diagnosis in homeless persons and shelter workers, even in major disease-free Brazilian or other worldwide cities, mostly due to early exposure and vulnerable living conditions.

## 1. Introduction

Chagas disease, a protozoal anthropozoonosis caused by *Trypanosoma cruzi*, is mostly transmitted by feces and urine of infected triatomine bugs and is occasionally acquired by oral ingestion, transplacental congenital, and blood transfusion (1). Chagas disease has been included among the neglected diseases worldwide, with ~6–7 million people globally affected, with 20–30% cardiomyopathy and death rates (2). Originally restricted to rural endemic Latin American areas, Chagas cases have been since reported in several urban areas of the Americas including Canada as well as the African, Eastern Mediterranean, European, and Western Pacific countries, associated with environmental changes and increased human mobility (3). In Latin America, established endemic areas have covered 21 countries, with ~100 million exposed individuals (2). In Brazil, chronic and asymptomatic disease forms may have been historically underdiagnosed as only acute Chagas cases have mandatory notification by the Ministry of Health (4). Nevertheless, chronic Chagas estimative may reach ~1.2–4.6 million Brazilians, causing 6,000 deaths per year (5).

Persons experiencing homelessness have been recognized as the most vulnerable populations worldwide, along with incarcerated persons, immigrants, abusive families, and refugees (6). The higher morbidity and less life expectancy in persons experiencing homelessness than the general population may be related to social inequality, absence of settled home associated with migratory movements, drug addiction, and mental health disorders (7). Vulnerable populations have also been associated with Chagas disease in several countries, particularly due to triatomine exposure, injecting drugs, and transplacental transmission, leading to high levels of disability and early mortality (7, 8).

Despite persons experiencing homelessness may be highly exposed outdoors to triatomine bugs and present higher risks of blood-borne and vertical transmission (9), no study to date has assessed anti-*T. cruzi* antibodies in such a population. Accordingly, the present study aimed to assess anti-*T. cruzi* antibodies in persons experiencing homelessness and related shelter workers of São Paulo city, southeastern Brazil.

## 2. Methods

The study herein was approved by the Ethics Committee in Human Health at the Brazilian Ministry of Health (CAAE: 80099017.3.3004.0086, protocol number: 3.366.684). This was a cross-sectional comparative serological study on people experiencing homeless and related shelter workers of São Paulo city, São Paulo state, southeastern Brazil. Although initially considered a control group, shelter workers were also a vulnerable and low-income population, with past and present exposure to Chagas vectors and disease, with public health importance on workers' health and occupational risk. Blood sampling was conducted in August 2020, during the COVID-19 pandemic when São Paulo was a pandemic world epicenter city, at a major day-only public service shelter, located in the eastern subregion, with the second highest city homeless population with ~5,000 individuals in 2020 (10). The shelter daily provided ~600 breakfast and 800 lunch meals at the time, with ~90 healthcare and assistance professionals providing direct care to homeless persons, as previously described (11).

Homeless people and shelter workers were invited to voluntarily participate and sign an official agreement form. Blood samplings were then performed by cephalic puncture; serum was obtained after centrifugation, stored at −20°C, and tested by using a commercial kit (Wiener Chagatest ELISA recombinant v.3.0, Wiener Laboratories SAIC, Argentina), using six purified recombinant antigens targets, with 99% sensitivity and 96% specificity to detect anti-*T. cruzi* antibodies, registered and approved by the Brazilian Ministry of Health.

## 3. Results

Overall, three of 203 (1.5%) persons experiencing homelessness and two of 87 (2.3%) shelter workers were seropositive to anti-*T. cruzi* antibodies. The three seropositive homeless persons were men, from 30 to 60 years old, mostly (2/3; 66.6%) born or lived previously outside São Paulo city, and identified themselves as brown color and “mixed-race Brazilians” with elementary school as the level of education, current alcohol and tobacco use, and unemployment as a reason for becoming homeless. No injecting drug use and history of sexually transmitted infections (STI) were mentioned by any seropositive homeless person. One individual reported a dry cough and fever. The two seropositive shelter workers were also 30 to 60 years old, one man and one woman, identified themselves as Black and white, with higher and elementary education, respectively. The seropositive woman reported current use of alcohol, tobacco, and cocaine, history of syphilis and hepatitis infection, chest pain, dry cough, and headache.

## 4. Discussion

To the authors' knowledge, this was the first worldwide serosurvey of *T. cruzi* in persons experiencing homelessness and correspondent shelter workers. Although the herein seropositivity in persons experiencing homelessness (1.5%) and shelter workers (2.3%) was within 0 to 25.1% seroprevalence for chronic Chagas disease in the general Brazilian population, overall the disease has decreased from 4.4% in the 1980s to 2.4% after 2000s and such a wide range may not reflect the local disease status (12). In addition, although the Chagas disease infection has been estimated in 1.0 to 2.4% of the Brazilian population (~1.9 to 4.6 million people) (13), acute cases of Chagas disease were reported in only 2/62 (3.2%) microregions of São Paulo state since 2008, with a relative risk of 0.01 in the São Paulo microregion in 2017 (14). Thus, in addition to potential autochthonous cases, such higher prevalence observed in the present study may be likely associated with socially vulnerable conditions and migration movements, as already established (8, 15).

As chronic Chagas disease has been mostly asymptomatic and based on the serological diagnosis, prevalence may be underestimated (7, 13). Nonetheless, Chagas disease was the main cause of death in the last 2 decades due to neglected tropical diseases in Brazil, with approximately a third of deaths occurring in a different state from the birthplace (16). In addition, individuals from other endemic Latin American countries may be infected before migration, as 28/633 (4.4%) Bolivian immigrants living in downtown São Paulo city were seropositive, epidemiologically associated with early rural laboring and known infected relatives in Bolivia (17).

As human migration has been indicated as a potential risk factor for Chagas disease, a Latin American problem may have turned into a global public health issue (18), particularly during the adaptation period, as migrants and refugees may likely experience vulnerability and homelessness. Not surprisingly, migrant groups stratified by birth country have commonly presented higher seropositivity when compared with nationwide endemicity (18). In such a scenario, human migrations have been indicated as the critical factor for Chagas disease emergence and spreading in disease-free areas, mostly transmitted even in the absence of vector transmission by blood transfusion, unprotected sexual life and pregnancy leading to vertical infection, and organ transplantation (19).

In the study herein, while shelter workers were mostly born in the São Paulo city (61.2%), homeless persons were mainly (68.0%) from other Brazilian cities, reflecting the well-described migratory phenomena of vulnerable populations to major urban centers (11). The authors hypothesized that the similar seroprevalence and exposure between homeless persons and shelter workers herein may be more associated with shared past and present low-income social vulnerability than migratory movements, which may also include infection by sharing injecting drugs, vertical transmission, or blood transfusion. However, as no epidemiological questions were made to shelter workers about temporary housing or visits to endemic areas outside São Paulo city, further studies should be conducted to fully establish such epidemiological findings. The results of this study corroborated the findings of shared vulnerability between homeless and related healthcare shelter workers during the COVID-19 pandemic (11). As previously suggested, healthcare workers in both globally high- and low-resource settings were likely to face risks and vulnerabilities that were shaped by local social and health system factors (20). In addition, non-healthcare workers, including shelter workers in cleaning, cooking, and maintenance services herein, may be also exposed to particular zoonotic pathogens and infectious bioaerosols at the workplace, as already shown (21).

Interestingly, the entomological surveillance service at São Paulo city has reported several confirmed vectors within city limits including *Panstrongylus geniculatus, Panstrongylus megistus, Triatoma infestans*, and *Triatoma sordida* infected by *T. cruzi* from 1988 to 2007 (22). Although these triatomine species were reported in the southern city area near the Atlantic Rainforest fragments, with no reports of vector domiciliation (22), *P. megistus* was recently reported in an urban São Paulo city park surrounded by artificial ecotopes (23). In addition, 34/108 (31.5%) triatomine species were molecularly infected by *T. cruzi* from 2012 to 2017 in the São Paulo state, with only one statewide reported case in 2016, two in 2017, and none in 2018, 2019, and 2020 (24). In this surveillance, the feeding behavior of collected triatomine species has shown contact with birds, opossum, rodents, and human beings (24). As triatomine species have been reportedly adapted to both natural and domestic areas (25), vulnerable populations may be more exposed outdoors due to absence of settled home or risk at work, neither ruling out nor confirming that positive homeless persons and shelter workers reported herein may be autochthonous cases of São Paulo city. As no molecular follow-up was made to characterize and compare strains of positive persons and those found in local vectors, we can only hypothesize on the matter. Thus, future studies are needed to confirm the active transmission of Chagas disease in São Paulo city, considering entomological and molecular surveys of triatomine species for *T. cruzi* infection, along with occurrence of acute cases in human populations and molecular matching of strains.

As limitations, the relatively low sampling and overall seropositivity herein may generate insufficient data to provide a representative statistical description and analysis of associated risk factors. In addition, the sampled homeless and shelter work populations were from a single city region with no bug vector report and may not be extrapolated to the entire homeless and healthcare populations and environmental exposure of São Paulo city. As no autochthonous case was confirmed in São Paulo city to date, negative results were expected, and the prevalence of general population was used as a control. As a cross-sectional approach, the present study represents a picture of people experiencing homeless and related shelter workers in São Paulo city in August 2020 for Chagas disease exposure. Samplings were performed in August 2020 only due to COVID-19, when São Paulo was the pandemic world epicenter city; otherwise, city hall would not allow samplings, and homeless persons and shelter workers would not come for samplings. Despite being required as ideal for research, control group sampling at the time was denied and later on would not reflect the two exposed groups. Nonetheless, further studies need to include a non-shelter worker or experiencing homelessness control group with the same sociodemographic conditions. In addition, a larger and randomized sample size of groups, along with better geographic representation, particularly in areas with the confirmed environmental presence of infected vectors, genetically compared *T. cruzi* strains were found in vectors, animals, and infected persons.

In conclusion, this is the first *T. cruzi* serosurvey in persons experiencing homelessness worldwide, with 3/203 (1.5%) seropositive homeless persons and 2/87 (2.3%) shelter workers, with similar seroprevalence likely associated with their previous and current low-income social vulnerabilities. As several *T. cruzi*-infected vectors have been described within São Paulo city limits, seropositive homeless persons and city-born shelter workers reported herein can neither be ruled out nor confirmed as autochthonous Chagas cases. Moreover, Chagas disease should be always considered as a differential diagnosis in homeless persons and shelter workers, even in major disease-free Brazilian or other worldwide cities, mostly due to early exposure and vulnerable living conditions.

## Data availability statement

The original contributions presented in the study are included in the article/supplementary material, further inquiries can be directed to the corresponding author.

## Ethics statement

The studies involving human participants were reviewed and approved by Ethics Committee in Human Health at the Brazilian Ministry of Health (CAAE: 80099017.3.3004.0086, protocol number: 3.366.684). The patients/participants provided their written informed consent to participate in this study.

## Author contributions

LBK, FBF, and AWB: conceptualization, writing the original draft, reviewing, and editing. LBK, ACC, and AWB: performed the samples collection. LBK and GG: performed the serological analyses. FBF: supervision. All authors have read and agreed to the final version of the manuscript.
